# Comparison of the diagnostic values of circulating steroid hormones, VEGF-A, PIGF, and ADAM12 in women with ectopic pregnancy

**DOI:** 10.1186/1479-5876-11-44

**Published:** 2013-02-19

**Authors:** Shien Zou, Xin Li, Yi Feng, Shan Sun, Jin Li, Emil Egecioglu, Håkan Billig, Ruijin Shao

**Affiliations:** 1 Department of Gynecology, Obstetrics and Gynecology Hospital of Fudan University, 200011, Shanghai, China; 2 Department of Physiology/Endocrinology, Institute of Neuroscience and Physiology, The Sahlgrenska Academy, University of Gothenburg, 40530, Gothenburg, Sweden; 3 Department of Integrative Medicine and Neurobiology, State Key Lab of Medical Neurobiology, Shanghai Medical College and Institute of Acupuncture Research (WHO Collaborating Center for Traditional Medicine), Institute of Brain Science, Fudan University, 200032, Shanghai, China; 4 Laboratory Center, Eye and Ear, Nose, Throat Hospital of Fudan University, 200031, Shanghai, China; 5 Institute of Bioscience, Fudan University, 200032, Shanghai, China

**Keywords:** Steroid hormones, VEGF-A, PIGF, ADAM12, Hypoxia, Tubal ectopic pregnancy

## Abstract

**Background:**

Several peripheral proteins that might be useful for detecting the presence of ectopic pregnancy (EP) have been evaluated, but none have been proven entirely useful in the clinic. We investigated the presence and the possible changes in circulating molecules that distinguish between normal intrauterine pregnancy (IUP) and tubal ectopic pregnancy.

**Methods:**

Non-pregnant women during the menstrual cycle, women with IUP, and women with tubal EP after informed consent. Serum levels of 17β-estradiol (E2), progesterone (P4), testosterone (T), beta-human chorionic gonadotropin (β-hCG), vascular endothelial growth factor-A (VEGF-A), placental growth factor (PIGF), and a distintegrin and metalloprotease protein 12 (ADAM12) were analyzed. Receiver operating characteristic analysis was used to assess the diagnostic discrimination of EP and gestational age-matched IUP.

**Results:**

E2, P4, PIGF, and ADAM12 levels increased and β-hCG decreased throughout IUP. E2 and VEGF-A levels were significantly different between women with tubal EP and IUP. However, using a serum β-hCG cut-off of less than 1000 mIU/mL, P4 was significantly lower in women with tubal EP compared to IUP. Although E2 was inversely correlated with VEGF-A in women in the early stages of IUP, E2 was not correlated with VEGF-A in women with EP prior to tubal surgery. There were no significant differences in either PIGF or ADAM12 alone between women with tubal EP or IUP. Although no significant correlations were seen between E2 and PIGF or P4 and ADAM12 in women in the early stages of IUP, E2 was positively correlated with PIGF and P4 was positively correlated with ADAM12 in women with EP prior to tubal surgery. Our studies defined associations but not causality.

**Conclusions:**

Individual measurements of serum E2 or VEGF-A levels are strongly related to early pregnancy outcomes for women with IUP and EP, and pregnancy-associated E2 and VEGF-A levels provide diagnostic accuracy for the presence of tubal EP. This study demonstrates that correlation analysis of E2/VEGF-A and E2/PIGF serum levels may be able to distinguish a tubal EP from a normal IUP.

## Background

Approximately 98% of ectopic pregnancies (EP) occur in the Fallopian tube [1]. The most significant complication of EP is rupture of the Fallopian tube causing massive internal bleeding, infection, and possibly death [2]. EPs are asymptomatic prior to rupture of the Fallopian tube [2,3] and at present the only way to conclusively diagnose EP is transvaginal ultrasound monitoring, preferably with β-human chorionic gonadotropin (β-hCG) and progesterone (P4) confirmation [3]. Although symptom-based diagnosis of tubal EP has improved [1,4], the diagnosis of EP is not always simple or straightforward. It has been reported that more than one-third of the women who have died from EP in the UK since 1997 had been mis-diagnosed [4]. Although several risk factors for tubal EP have been identified [5-8], the underlying mechanisms leading to tubal EP are not fully understood [1,9]. There is currently no accurate method to identify what treatments for tubal dysfunction should be undertaken to prevent EP [1,9]. Because an early and accurate laboratory diagnosis of tubal EP could assist clinical management, various laboratories have directed their research toward biochemical markers of tubal EP [10-13]. Several peripheral proteins that might be useful for detecting the presence of EP have been evaluated [14,15], but none have been proven entirely useful in the clinic.

The vascular endothelial growth factor (VEGF) family consists of VEGF-A (generally called VEGF), VEGF-B, VEGF-C, VEGF-D, VEGF-E, and placental growth factor (PIGF). These are angiogenic factors that participate in the regulation of early placental angiogenesis and maternal spiral artery remodeling [16]. A disintegrin and metalloprotease protein 12 (ADAM12) is one member of a disintegrin and metalloprotease protein family that possesses extracellular metalloprotease and cell-binding functions as well as intracellular signaling capacities [17]. Although the mechanisms underlying the functional roles of VEGF-A, PIGF, and ADAM12 in tubal EP are not clear, circulating levels of VEGF-A [12,18-23], PIGF [23-25], and ADAM12 [26-28] have been shown to change in women with EP. However, the retrospective case-control studies have yielded inconsistent results for such correlations [24,27,29]. The regulatory effects of changes in reproductive steroid hormones or of menstrual cycle phase on the circulating levels of VEGF-A, PIGF, and ADAM12 in women throughout normal intrauterine pregnancy (IUP) and post-term delivery are far less studied and understood. Furthermore, to our knowledge there are no studies making direct comparisons of these candidate protein biomarkers for EP diagnosis within the same cohort.

It has long been recognized that early diagnosis is important for making therapeutic decisions in regards to tubal EP that improve future fertility. Because the measurement of candidate biomarkers in the circulation is a noninvasive procedure and is relatively simple to perform without requiring special instruments and personnel, the development and validation of circulating biomarkers for the early diagnosis of tubal EP is warranted. The present study aimed to evaluate whether circulating hormone (17β-estradiol (E2), progesterone (P4), testosterone (T), and β-hCG), VEGF-A, PIGF, and ADAM12 levels vary in women during the menstrual cycle, in women with IUP, and in women with EP before and after tubal surgeries.

## Methods

### Ethics statement

This study was approved by the Ethics Committees of the Obstetrics and Gynecology Hospital and Shanghai Medical College, Fudan University, China. All participants provided informed consent.

### Study design and sample collection

All participants underwent clinical examination at the Obstetrics and Gynecology Hospital of Fudan University, Shanghai, China. Clinical work-up included menstrual history as well as current cycle length and menstrual regularity.

Exclusion criteria included use of estrogen- or progestin-containing medication within three months of the study, past EP, any gynecological pathology (e.g., endometriosis, fibroids, or any operation to the gynecological organs), infection, and smoking. All the patients whose pregnancies resulted from assisted reproductive technologies were also excluded from the study.

Blood samples were collected into Sarstedt evacuated tubes without anticoagulant. All blood samples were centrifuged at 1000× *g* for 15 min, and the serum was stored at −80 ° C until batch analyses. The present study included non-pregnant and pregnant women (total n = 153) who were subdivided into the following groups:

Group 1. The different stages of the menstrual cycle in non-pregnant women (n = 78) were studied. Blood samples were collected at the scheduled visits during their menstrual cycle. Menstrual cycle day was established using the criteria reported by Noyes et al. [30]. Sample dating characterized the samples as coming from the proliferative (days 1-14 of the cycle), early secretive (days 15-18), mid-secretive (days 19-23), and late secretive (days 24-28) phases of the menstrual cycle. Regular menstrual cycles were defined as an average cycle length of 26-30 days, with no more variation than ± 3 days from the average. Transabdominal ultrasonography was also performed to assess ovarian volume and uterine thickness.

Group 2. This group comprised healthy pregnant women studied longitudinally throughout gestation and post-term delivery (n = 42). Blood samples were collected at the scheduled visits during their IUP, and on days 1-3 after spontaneous labor and vaginal delivery. The diagnosis of a normal IUP was made upon the observation of an intrauterine gestational sac or a live embryo on the transvaginal or transabdominal ultrasound scan. Only women with normally progressing pregnancies were studied during their visits to the prenatal clinic during the early, middle, and late stages of their IUP.

Group 3. In this group, women with EP (n = 32) were studied before and after tubal surgery and matched to a subgroup of women with early IUP. A full medical history was documented, and clinical examination was carried out by the attending physician. Transvaginal ultrasonography was performed and the serum β-hCG levels were analyzed in patients at the time of their first clinical presentation. Blood samples were collected from patients at the time of surgery or 2-3 days after surgery. None of the women undergoing surgical management of EP presented with acute hemodynamic shock. Women with EP were diagnosed during laparoscopy and on histological examination of the surgical specimens.

### Main outcome measures

All sera were stored at −80°C before performing the assays, and aliquots that had not been previously thawed were used in the present study. Samples were tested in duplicate and analyzed individually. Radioimmunoassays (RIA) were performed at Beijing Free Co. (China), and enzyme-linked immunoassays (ELISA) were performed at the Department of Integrative Medicine and Neurobiology, Shanghai Medical College, Fudan University (China). The averages of the duplicate readings for each standard, control, and individual samples were used for the analyses.

#### E2, P4, T, and β-hCG assays

Serum E2 (with an assay sensitivity less than 5.0 pg/mL, an intra-assay coefficient of variation (CV) of 10.0%, and an interassay CV of 15.2%), P4 (with an assay sensitivity less than 5.0 ng/mL, an intra-assay CV of 5.0%, and an interassay CV of 10.0%), T (with an assay sensitivity less than 0.1 ng/mL, an intra-assay CV of 8.0%, and an interassay CV of 15.0%), and β-hCG (with an assay sensitivity less than 10.0 mIU/mL, an intra-assay CV of 5.0%, and an interassay CV of 10.0%) levels were measured by competitive RIA (^125^I - Kit, Beijing Free Co.) using direct-coated tube technology. E2, P4, T, and β-hCG were labeled with ^125^I as the tracer, and known quantities of unlabeled E2, P4, T, and β-hCG were used to construct standard curves. The concentrations used for the standard curves were 0-4000 pg/mL for E2, 0-100 ng/mL for P4, 0-10 ng/mL for T, and 0-1600 mIU/mL for β-hCG.

#### VEGF-A, PIGF, and ADAM12 assays

All samples were brought to room temperature and vortexed for 5 s before applying them to the ELISA plate. Serum levels of VEGF-A (with an assay sensitivity less than 5.0 pg/mL, an intra-assay CV of 6.7%, and an interassay CV of 8.8%), PIGF (with an assay sensitivity less than 7.0 pg/mL, an intra-assay CV of 7.0%, and an interassay CV of 11.8%), and ADAM12 (with an assay sensitivity less than 0.03 ng/mL, an intra-assay CV of 4.1%, and an interassay CV of 5.5%) were measured by ELISA. The ELISA kits for the three proteins were catalog numbers DVE00, DPG00, and DAD120, respectively, from R&D Systems, Inc., Minneapolis, MN. Assays were performed as described previously [21,25,27] according to the manufacturer’s instructions and with reagents and materials provided by the manufacturer.

### Statistical analysis

Numerical, grouped results are expressed as the mean ± SEM. In all analyses, a P value less than 0.05 was considered statistically significant. Statistical analysis was performed using SPSS version 16.0 for Windows (SPSS Inc., Chicago, IL). Comparison among the various groups was performed using nonparametric tests (Kruskal-Wallis followed by multiple comparison procedures according to Dunn’s method) because our variables did not have a normal distribution. Correlation between variables was performed using Spearman’s analysis.

The specificity and sensitivity of the various assays as diagnostic tests were assessed using receiver operating characteristic (ROC) curve analysis [31]. As opposed to accuracy, sensitivity and specificity are not dependent on the prevalence of the disease in the sample. Thus, ROC curve analysis provides a description of disease detectability that is independent of both disease prevalence and decision threshold effects. For ROC analysis, women with EP were considered affected, and IUP as unaffected. ROC curves were constructed by plotting the sensitivity (true-positive) on the ordinate as a function of the complement of specificity (false-positive) for all possible cut-off values of the diagnostic test [32]. Greater deviation toward the left upper corner of the curve indicates better detection of EP.

## Results

The demographics and laboratory characteristics of the normal menstrual cycle women and those with IUP and EP are shown in Table 1 and 2. There were no significant differences in age between the three groups and there were also no significant differences in the number of gestational days in women with EP and early IUP. Consequently, we examined the relationships between hormones and VEGF-A, PIGF, and ADAM12 in these groups using unadjusted Spearman’s analysis (Tables 3, 4 and 5).

**Table 1:** Patient characteristics and hormonal profiles

	**n**	**Age**	**GA**	**E2**	**P4**	**E2/P4**	**T**	**β-hCG**
		**(years)**	**(days)**	**(pg/mL)**	**(ng/mL)**		**(ng/mL)**	**(mIU/mL)**
**Menstrualcycle phase**								
Proliferative	30	25.90 ± 0.50	-	37.99 ± 4.76	0.90 ± 0.12	0.06 ± 0.012	0.21 ± 0.02	-
Early secretive	13	25.85 ± 0.55	-	16.84 ± 4.52	2.59 ± 0.82	0.01 ± 0.004^▲^	0.15 ± 0.02	-
Mid secretive	20	25.10 ± 0.40	-	14.57 ± 4.15	5.05 ± 1.02	0.02 ± 0.008^▲▲^	0.15 ± 0.05	-
Late secretive	15	25.80 ± 1.09	-	14.83 ± 2.97	3.80 ± 1.15	0.01 ± 0.003^▲▲^	0.15 ± 0.04	-
**Intrauterine pregnancy**								
Early	14	26.36 ± 1.46	50.57 ± 1.36	176.61 ± 21.87	13.00 ± 1.65	0.02 ± 0.003	0.53 ± 0.05	21320.76 ± 1700.47
Middle	10	27.33 ± 1.03	115.50 ± 4.06	211.91 ± 42.04	15.81 ± 1.03	0.01 ± 0.003	0.27 ± 0.05	17727.61 ± 2925.63
Late	13	27.58 ± 1.08	255.11 ± 8.16	499.62 ± 87.98^###^	89.93 ± 11.99 ^###^	0.01 ± 0.003	0.70 ± 0.08	12311.79 ± 1644.37^##^
Post-term delivery	10	30.70 ± 1.27	-	39.86 ± 5.35	6.62 ± 0.74	0.01 ± 0.001	0.37 ± 0.04	3691.29 ± 816.94 ^###^
**Ectopic pregnancy**								
Before surgery								
β-hCG< 1,000mIU/mL	17	29.06 ± 1.13	48.00 ± 4.31	36.09 ± 5.00	3.65 ± 0.77	0.02 ± 0.003	0.41 ± 0.05	813.45 ± 350.12
β-hCG ≥ 1,000mIU/mL	15	31.93 ± 1.56	47.20 ± 3.87	33.31 ± 6.45	6.97 ± 1.30	0.01 ± 0.008^*^	0.48 ± 0.04	6505.13 ± 1999.57
Total	32	30.41 ± 0.96	47.61 ± 2.86	34.70 ± 4.01	5.37 ± 0.82	0.02 ± 0.004	0.45 ± 0.03	3573.05 ± 1092.31
After surgery								
β-hCG< 500mIU/mL	16	29.13 ± 1.28	-	54.53 ± 15.90	2.04 ± 0.38	0.03 ± 0.006^*^	0.37 ± 0.06	237.15 ± 36.15
β-hCG ≥ 500mIU/mL	6	29.50 ± 1.73	-	26.03 ± 9.77	2.43 ± 0.38	0.01 ± 0.003 ^*^	0.26 ± 0.07	1792.07 ± 353.56
Total	22	29.23 ± 1.02	-	46.75 ± 12.05	2.14 ± 0.29	0.02 ± 0.005	0.34 ± 0.05	703.63 ± 192.92

GA, gestational age. Data are presented as mean ± SEM. Statistical analysis was performed using SPSS version 16.0 for Windows (SPSS Inc., Chicago, IL).

A nonparametric, unpaired test (Kruskal-Wallis test) followed by Dunnett's*post hoc* test was used for comparison of continuous variables.

▲ p<0.05, ▲▲ p<0.01 vs. the proliferative phase of the endometrial cycle; ## p<0.01, ### p<0.001 vs. the early stage of intrauterine pregnancy;

* p<0.05 vs. ectopic pregnancy (before surgery, β-hCG< 1,000mIU/mL).

**Table 2:** Patient VEGF-A, PIGF, and ADAM12 levels

	**n**	**VEGF-A**	**PIGF**	**ADAM12**
		**(pg/mL)**	**(pg/mL)**	**(ng/mL)**
**Menstrualcycle phase**				
Proliferative	30	420.74±31.75	49.64±1.12	0.94±0.06
Early secretive	13	391.59±50.64	49.16±3.52	1.41±0.13
Mid secretive	20	389.00±43.50	64.08±8.32	1.19±0.15
Late secretive	15	284.57±26.72	79.95±10.05	0.43±0.04
**Intrauterine pregnancy**				
Early	14	169.29±59.77	43.05±1.06	2.35±0.70
Middle	10	91.33±3.30	363.42±160.63^###^	20.43±3.97
Late	13	95.13±4.68	517.87±85.97^###^	83.12±13.96^###^
Post-term delivery	10	661.67±192.11	46.22±1.30	41.81±4.49^###^
**Ectopic pregnancy**				
Before surgery				
β-hCG< 1,000mIU/mL	17	655.76±56.30	43.88±1.11	1.35±0.24
β-hCG ≥ 1,000mIU/mL	15	1095.56±185.03	41.03±1.49	1.61±0.29
Total	32	853.67±99.62^**^	42.60±0.94	1.46±0.18
After surgery				
β-hCG< 500mIU/mL	16	1080.63±150.38	44.99±1.29	1.33±0.06
β-hCG ≥ 500mIU/mL	6	1584.67±234.67	46.31±1.18	1.42±0.06
Total	22	1200.63±133.60^***^	45.30±1.02	1.35±0.05

Data are presented as mean ± SEM. Statistical analysis was performed using SPSS version 16.0 for Windows (SPSS Inc., Chicago, IL). A nonparametric, unpaired test (Kruskal-Wallis test) followed by Dunnett's*post hoc* test was used for comparison of continuous variables. ### p<0.001 vs. the early stage of intrauterine pregnancy; ** p< 0.01, *** p< 0.001 vs. ectopic pregnancy (before surgery, β-hCG< 1,000).

**Table 3:** Correlation between VEGF-A level and hormone concentrations

	**E2**	**P4**	**T**	**β-hCG**
**Endometrial cycle phase**				
Mid secretive	−0.08 (0.75)	0.05 (0.83)	−0.25 (0.30)	n.d.
**Intrauterine pregnancy**				
Early	**−0.67** (0.01)	−0.19 (0.53)	−0.29 (0.34)	0.14 (0.65)
Post-term delivery	0.42 (0.27)	−0.18 (0.64)	−0.42 (0.26)	−0.53 (0.12)
**Ectopic pregnancy**				
Before surgery	0.06 (0.80)	−0.25 (0.29)	**0.46** (0.04)	−0.06 (0.85)
After surgery	**−0.43** (0.05)	−0.29 (0.20)	−0.34 (0.13)	0.36 (0.13)

The results are shown as the r-value of the Spearman correlation at p < 0.01 andthe 95% confidence interval. The significance (P value) of the correlation is in parentheses and boldface values are statistically significant.

*n.d*., not determined.

**Table 4:** Correlation between PIGF level and hormone concentrations

	**E2**	**P4**	**T**	**β-hCG**
**Endometrial cycle phase**				
Mid secretive	−0.17 (0.48)	0.10 (0.69)	0.04 (0.87)	n.d.
**Intrauterine pregnancy**				
Early	−0.01 (0.99)	0.48 (0.10)	−0.29 (0.34)	−0.06 (0.85)
Post-term delivery	0.25 (0.52)	0.13 (0.73)	−0.25 (0.52)	−0.23 (0.52)
**Ectopic pregnancy**				
Before surgery	**1.00** (<0.001)	−0.13 (0.57)	−0.30 (0.19)	−0.04 (0.87)
After surgery	**−0.48** (0.03)	−0.11 (0.65)	**−0.44** (0.05)	0.09 (0.71)

The results are shown as the r-valueof the Spearman correlation at p < 0.01 and a 95% confidence interval. The significance (P value) of the correlation is in parentheses and boldface values are statistically significant.

*n.d*., not determined.

**Table 5:** Correlation between ADAM12 level and hormone concentrations

	**E2**	**P4**	**T**	**β-hCG**
**Endometrial cycle phase**				
Mid secretive	−0.33 (0.15)	−0.23 (0.33)	0.11 (0.63)	n.d.
**Intrauterine pregnancy**				
Early	0.36 (0.22)	−0.34 (0.26)	0.53 (0.06)	0.07 (0.82)
Post-term delivery	0.12 (0.77)	−0.43 (0.24)	0.38 (0.31)	0.18 (0.63)
**Ectopic pregnancy**				
Before surgery	−0.13 (0.57)	**1.00** (<0.001)	0.15 (0.54)	−0.31 (0.18)
After surgery	**−0.45** (0.04)	−0.20 (0.40)	−0.36 (0.11)	0.19 (0.43)

The results are shown as the r-value of the Spearman correlation at p < 0.01 anda 95% confidence interval. The significance (P value) of the correlation is in parentheses and boldface values are statistically significant.

*n.d*., not determined.

### The menstrual cycle

Although a nonparametric, unpaired Kruskal-Wallis test did not show significant differences between the four endometrial cycle phases, serum E2 and P4 levels exhibited characteristic changes during the menstrual cycle. The E2:P4 ratio was significantly decreased in the early-, mid-, and late-secretive phases compared to the proliferative phase (Table 1). However, no significant differences in serum T, VEGF-A, PIGF, or ADAM12 levels were observed between any of the phases (Table 1 and 2). There was no significant correlation between steroid hormones (E2, P4, and T) and candidate biomarkers (VEGF-A, PIGF, and ADAM12) in the mid-secretive phase (Tables 3, 4 and 5).

### IUP

Although serum T levels were not significantly different between early, middle, and late stages of IUP and post-term delivery, serum E2 and P4 levels increased progressively during these stages, peaked in the late stage of IUP, and fell after delivery. Serum β-hCG levels were high in the early stage of IUP and decreased throughout IUP and post-term delivery. Serum PIGF and ADAM12 levels also became progressively higher throughout the course of IUP, and were highest in the late stage of IUP (Table 2). Serum levels of PIGF and ADAM12 decreased after delivery. No significant changes in the E2:P4 ratio or serum T and VEGF-A levels were observed (Table 1 and 2), but there was a significant correlation between serum E2 and VEGF-A levels in the early stages of IUP (r = −0.67, P < 0.01) (Table 3).

### Differences between IUP and the mid-secretive phase of the menstrual cycle

**Figure 1:**
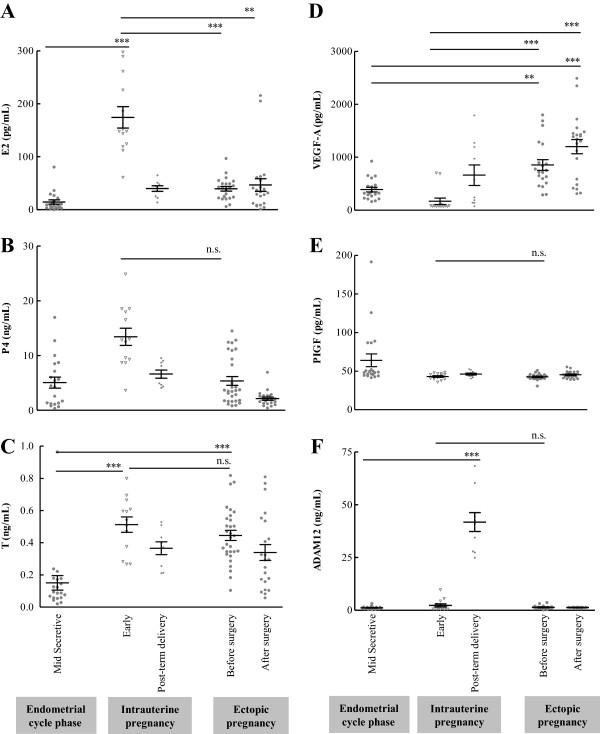
**Serum levels of E2, P4, T, VEGF, PIGF, and ADAM12 in women with IUP divided into early IUP and post-term delivery subgroups, and in women with EP before and after tubal surgeries compared with women in the mid-secretive phase of the endometrial cycle.** All data are expressed as the mean ± SEM. A nonparametric, unpaired test (Kruskal-Wallis test) followed by Dunnett's post-hoc test was used for multiple comparisons of continuous variables. ** P<0.01, and *** P<0.001. n.s., not significant.

Serum E2 and T levels were significantly higher in the early stage of IUP than in the mid-secretive phase of the menstrual cycle (Figure 1A and C). Serum ADAM12 levels were also significantly higher in women with normal IUP post-term delivery than in women in the mid-secretive phase of the menstrual cycle (Figure 1F).

### Tubal EP

**Figure 2:**
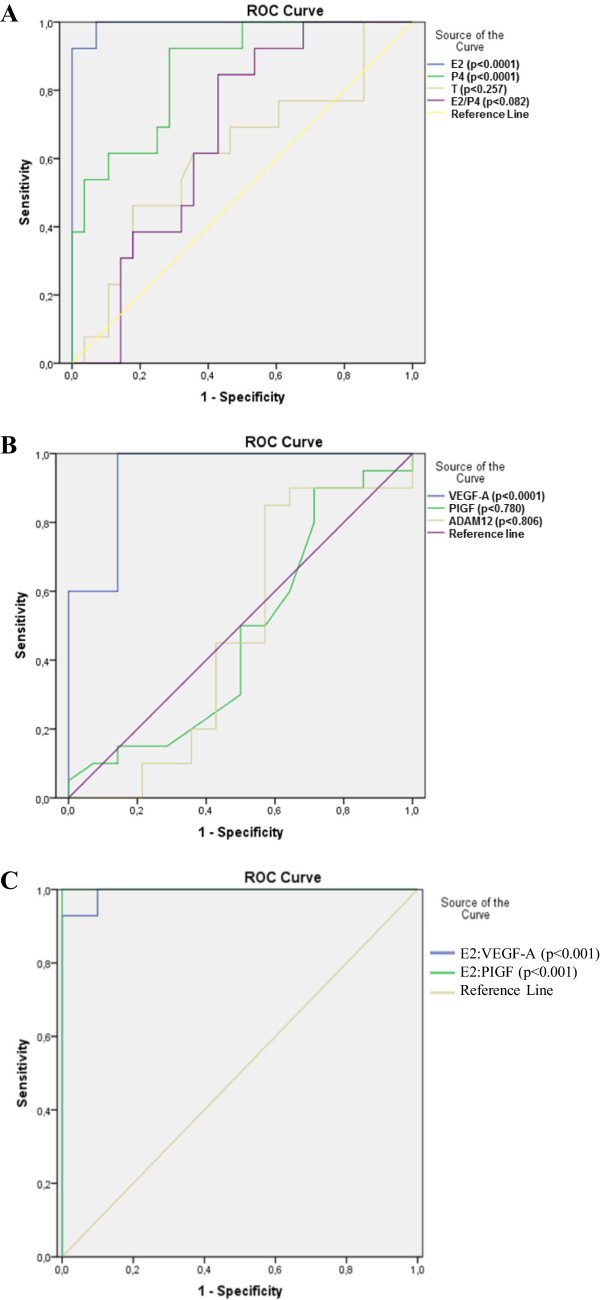
**(A) E2, P4, T levels and the E2:P4 ratio as diagnostic tests for women with tubal EP were assessed by the receiver operating curve (ROC) test.** ROC analysis was performed with SPSS version 16.0 for Windows, and statistical significance (P value) is indicated. The area under the curve was 0.99 (95% CI: 0.98-1.00) for E2, 0.86 (95% CI: 0.75-0.98) for P4, 0.61 (95% CI: 0.42-0.80) for T, and 0.67 (95% CI: 0.51-0.83) for the E2:P4 ratio. (**B**) VEGF, PIGF, and ADAM12 as diagnostic tests for women with tubal EP were assessed by the ROC test. The area under the curve was 0.94 (95% CI: 0.86-1.00) for VEGF, 0.47 (95% CI: 0.26-0.68) for PIGF, and 0.48 (95% CI: 0.25-0.69) for ADAM12. (**C**) The E2: VEGF-A and E2: PIGF ratio as diagnostic tests for women with tubal EP were assessed by the ROC test. The area under the curve was 0.99 (95% CI: 0.97-1.00) for the E2: VEGF-A ratio, and 1.00 (95% CI: 1.00-1.00) for the E2: PIGF ratio.

Table 1 lists the variations in the serum β-hCG levels in women with EP before and after tubal surgeries. Significant differences in the E2:P4 ratios were only observed when using two different cut-off values (500 and 1000 mIU/mL, Table 2). Because the variations in the serum concentrations of E2, P4, T, VEGF-A, PIGF and ADAM12 did not differ in women with EP with different β-hCG levels within the same surgical group, the results of the two EP subgroups before or after tubal surgery are presented as a total group (Tables 3, 4 and 5, Figure 1 and 2). In contrast to women with EP prior to tubal surgery, serum E2 levels were correlated with serum VEGF-A and ADAM12 levels after tubal surgery (r = −0.43, P = 0.05 (Table 3) and r = −0.45, P = 0.04 (Table 5), respectively). Serum E2 levels were significantly correlated with serum PIGF levels in women with EP both before (r = 1.00, P < 0.001) and after (r = −0.48, P = 0.03) tubal surgeries (Table 4). In contrast to E2, serum P4 levels were significantly correlated with serum ADAM12 levels in women with EP prior to tubal surgery (r = 1.00, P < 0.001) (Table 5). Serum T levels correlated well with serum VEGF-A levels in women with EP prior to tubal surgery (r = 0.46, P = 0.04) (Table 3) and with serum PIGF levels after tubal surgery (r = −0.44, P = 0.05) (Table 4).

### Differences between tubal EP and the mid-secretive phase of menstrual cycle

Serum T levels were significantly higher in women with EP before tubal surgery than in women in the mid-secretive phase of the menstrual cycle (Figure 1C). Serum VEGF-A levels were significantly higher in women with EP both before and after tubal surgeries than in women in the mid-secretive phase of the menstrual cycle (Figure 1D).

### Differences between tubal EP and gestational age-matched IUP

Women with EP both before and after tubal surgeries had lower levels of E2 than the gestational age-matched women with IUP (Figure 1A). The mean difference in E2 levels between tubal EP before surgery and the early stage of IUP was 139.73 pg/mL (95% CI: 95.14-184.31, P < 0.001). There were no significant differences in serum P4 levels in tubal EP compared with the early stage of IUP (Figure 1B). However, further analysis with a serum β-hCG cut-off level (<1000 mIU/mL) showed a significant difference in P4 levels between women with tubal EP with less than 1000 mIU/mL β-hCG and women in the early stage of IUP (3.65 ± 0.77 vs. 13.00 ± 1.65 mIU/mL, P < 0.001). The levels of serum β-hCG were significantly lower in women with tubal EP than in women in the early stage of IUP (Table 1). Women with EP both before and after tubal surgeries had higher levels of VEGF-A than in the gestational age-matched women with IUP (Figure 1D). The mean difference in VEGF-A levels between tubal EP prior to surgery and the early stage of IUP was 684.38 pg/mL (95% CI: 420.54-948.22, P < 0.001). However, no significant differences in T, PIGF, or ADAM12 levels alone were observed between women with tubal EP and gestational age-matched women with IUP (Figures 1C, E and F).

The diagnostic accuracy of the various parameters (E2, P4, T, VEGF-A, PIGF, and ADAM12 levels and the E2:P4 ratio) in the tubal EP and early IUP groups was evaluated by ROC analysis (Figures 2A and B). The area under the ROC curve represents the probability of correctly distinguishing between gestational age-matched women with tubal EP or IUP. The areas under the curves for serum E2, P4, and VEGF-A levels for the diagnosis of tubal EP were 0.99 (95% CI: 0.98-1.00, P < 0.0001), 0.86 (95% CI: 0.75-0.98, P < 0.0001), and 0.94 (95% CI: 0.86-1.00, P < 0.0001), respectively. However, the areas under the curves for serum T, PIGF, and ADAM12 levels for the diagnosis of tubal EP were 0.61 (95% CI: 0.42-0.80, P > 0.05), 0.47 (95% CI: 0.26-0.68, P > 0.05), and 0.48 (95% CI: 0.25-0.69, P > 0.05), respectively. These ROC curve analyses showed that the identification of women with tubal EP could not rely on measurements of serum levels of T, PIGF, or ADAM12 alone. In addition, the areas under the curves for the E2: VEGF-A and E2: PIGF ratios for the diagnosis of tubal EP were 0.99 (95% CI: 0.97-1.00, P < 0.001) and 1.00 (95% CI: 1.00-1.00, P < 0.001), respectively (Figure 2C).

## Discussion

There is a need for a sensitive and specific diagnostic biomarker that can facilitate early diagnosis and permit timely treatment of tubal EP [14,15]. Currently, the limited specificity and sensitivity of circulating biomarkers for the early diagnosis of tubal EP has greatly restricted their reliability.

The present study has significant strengths. First, this study shows in an independent and well-defined sample set that individual measurements of serum P4 levels were lower in women with tubal EP than gestational age-matched women with IUP, but this difference was not statistically significant. Sub-group analysis, however, provided a much more striking result. When samples were compared at a serum β-hCG level of less than 1000 mIU/mL, serum P4 levels differed significantly between the tubal EP and gestational age-matched IUP groups. These results suggest that using a combination of serum P4 and β-hCG levels can possibly differentiate a certain group of women with tubal EP from those with normal IUP. More importantly, we show that individual measurements of serum E2 or VEGF-A levels have significant advantages over serum P4 levels for distinguishing tubal EP from gestational age-matched IUP. Our data, together with that provided by others [15,33], show that circulating E2 levels are higher in women with tubal EP than non-pregnant women in the mid-secretive phase of the menstrual cycle, but are lower than in gestational age-matched women with IUP. These findings indicate that abnormal E2 levels may interrupt the tubal microenvironment and ultimately lead to improper embryo implantation in the Fallopian tube. In line with previous studies [12,18-23]despite differences in the study populations, we have found a significant induction in VEGF-A levels in women with EP relative to gestational age-matched women with IUP. Using an increased cut-off of 200 pg/mL, we have shown that all women with tubal EP (n = 33) had a serum VEGF-A level more than 200 pg/mL, which is consistent with previous studies [18,21]. The data indicate that a serum VEGF-A level of greater than 200 pg/mL can be used to discriminate a tubal EP from a normal IUP in women. Additionally, we found that serum E2 and VEGF-A concentration measurements generated superior diagnostic accuracy. Taken together, these data suggest that E2 and VEGF-A can be useful biomarkers for the early diagnosis of tubal EP.

The second important finding of the present study is that there are different correlations between the levels of steroid hormones and VEGF-A, PIGF, and ADAM12 in both the early stage of IUP and in tubal EP. This suggests that their in vivo biological functions should not be considered independently of each other. We show that, in contrast to the early stage of IUP, serum E2 levels do not correlate with serum VEGF-A levels but do correlate with serum PIGF levels in tubal EP. We also show that serum T levels correlate with serum VEGF-A levels and that serum P4 levels correlate with serum ADAM12 levels in tubal EP. Although highly suggestive, these significantly correlated data cannot prove that steroid hormones are responsible for the changes in the levels of VEGF-A, PIGF, and ADAM12 in the Fallopian tube. Embryonic implantation in the uterus is a complex developmental process that is likely mediated by a variety of molecules such as steroid hormones, growth factors, and cytokines [34]. However, little is known about the interactions of these molecules, especially in terms of tubal implantation [5,9]. Previous studies have demonstrated the use of combinations of circulating proteins for the diagnosis of EP [12,22], and we show here that the E2: VEGF-A and E2: PIGF ratios can aid in distinguishing tubal EP from normal IUP. It will be of interest in future studies to validate the results from our study with additional cohorts and to determine whether alternative combinations of these markers may demonstrate improved performance.

### Possible mechanisms responsible for the regulation of VEGF/PIGF expression, secretion, and signaling pathways during the development of tubal EP

To grow to the point of causing harm, a tubal EP requires the development of a local blood supply and angiogenesis at the tubal implantation site. It is thus reasonable to expand upon clinical research on circulating VEGF isoforms to understand the mechanisms by which their synthesis is regulated and to understand their biological functions during Fallopian tubal implantation. Such research would allow for a better understanding of the actual diagnostic values of VEGF-A and PIGF. In the human Fallopian tube, VEGF is localized in the epithelial cells, smooth muscle cells, and blood vessel cells in a region-specific manner [35,36], and significantly higher levels of tubal VEGF and VEGF receptor mRNAs are detected in women with a hydrosalpinx [37], which is defined as tubal dilation and abnormal fluid accumulation [6]. Moreover, in vitro studies have shown an increase in VEGF and soluble VEGF receptor secretion in human tubal epithelial cells and stromal fibroblasts in response to hypoxic stimulation [38]. It has been shown that VEGF-A and VEGF receptor mRNAs are significantly increased in the implantation site compared to non-implantation sites of human Fallopian tubes [39], and these are associated with trophoblastic invasion into the tubal wall in vivo [40] suggesting that the development of tubal EP could contribute to the elevation of circulating VEGF-A levels. Moreover, ligation of the Fallopian tube, which mimics the tubal occlusion that likely induces an EP in women, increases VEGF protein expression in rat Fallopian tubes [41].

Our results, together with those from other laboratories, provide a basis for proposing a working model for regulation and activation of VEGF isoforms and their receptor signaling pathways during tubal implantation. Disruption of the local environment such as a low oxygen level, possibly due to embryo implantation in the Fallopian tube, induces elevated levels of hypoxia-inducible factors (HIFs) [42]. This leads to tubal VEGF-A synthesis and secretion resulting in the abnormal levels of circulating VEGF-A seen in women with EP. HIF, a critical hypoxia sensor, is a heterodimeric complex composed of three alpha subunits (HIF-1α, HIF-2α, and HIF3α) and a stable beta subunit (HIF-1β, also known as aryl hydrocarbon receptor nuclear translocator (ARNT)) [42]. Hypoxic HIF activity is controlled primarily through post-translational modification and stabilization of the HIF-1α and HIF-2α subunits, and HIF-1β expression levels constitute important determinants of hypoxia responsiveness [42]. It is well known that heterodimeric complexes of HIF-1α/β translocate to the nucleus and activate several hypoxia-associated genes, including VEGF [16]. VEGF-A can form homodimers with itself and/or heterodimers with PIGF, and acts by binding to its two cell surface tyrosine kinase receptors, FLT-1 (VEGFR1) and KDR/FLK-1 (VEGFR2). PIGF binds only to FLT-1 (VEGFR1) [16]. Thus, implantation in the Fallopian tube leads to the coordinated activation of a transcriptional cascade in response to the presence of excessive hypoxia. As a result, tubal VEGF-A levels are increased and this activates the VEGF signaling cascades in an autocrine manner. On the other hand, increased levels of tubal VEGF causes them to be released into the circulation, and this leads to activation of the VEGF signaling cascades in a paracrine manner. Both autocrine and paracrine regulation may result in tubal fluid secretion, vascular defects, angiogenic dysfunction, and tubal wall damage. During normal pregnancy, VEGF isoforms are involved in building the placenta [16]. VEGF-A is expressed in the human placenta throughout gestation [43], and placenta-specific PIGF has a similar role as VEGF during normal IUP [16]. We show that serum PIGF levels are significantly increased in the middle and late stages of IUP compared to the early stage of IUP. In contrast to VEGF-A, however, we were unable to find a significant difference in serum PIGF levels between women with tubal EP and gestational age-matched women with IUP. Some previous studies have indicated that serum PIFG levels are elevated in tubal EP compared to IUP [23,24]. This discrepancy may be due to the different ethnic backgrounds of the patients analyzed, the pooling of blood samples from different gestational-aged women with acute and chronic clinical presentation of EP, and the use of a non-gestational age-matched IUP as a comparison control.

E2 has traditionally been considered the major hormonal regulator of the Fallopian tube [34]. E2 exerts its biological effects by binding to the estrogen receptor (ER), which exists as two different subtypes, ERα and ERβ. Both ER subtypes are expressed in normal human Fallopian tubes [5,44-46]. Moreover, ERα is frequently lost in the implantation and non-implantation site (our unpublished data) of the Fallopian tube in women who have suffered from EP [44,47]. Although the VEGF gene promoter harbors the estrogen response element [47], whether E2 is able to directly regulate VEGF-A expression via the activation of ER signaling in human Fallopian tubes is not fully understood. On the other hand, animal studies suggest that E2 and hypoxia can cooperate to regulate the same target in the Fallopian tube. For example, the expression of erythropoietin, a potent anti-inflammatory cytokine, is increased by both E2 and hypoxia in mouse Fallopian tubes both in vivo and in vitro [48]. Treatment with E2 followed by hypoxic stimulation significantly reduces VEGF-A protein synthesis and release in human endometrial tissues in vitro [49]. Because the C-terminal domain of HIF-1β, a potent coactivator of ER-dependent transcription, is essential for the enhancement of ER transcription [50], E2-dependent regulation of VEGF expression and secretion may be indirect through HIF isoforms during the Fallopian tubal implantation. Furthermore, in vitro experiments have shown that insulin-like growth factor-1 and interleukin-1β directly regulate VEGF and VEGFR expression in human tubal epithelial cells and stromal fibroblasts [51,52]. Collectively, these observations show that different molecules contribute to the regulation of VEGF synthesis and secretion, but their precise roles in tubal EP are not clearly understood. No existing mouse models can establish causative roles for factors implicated in the pathogenesis of EP [1,9], thus new animal models will need to be developed to specifically address these issues.

Although placenta-specific ADAM12 has been previously reported to distinguish EP from IUP [12,26], our findings are in agreement with a recent finding by Horne et al. [27] that demonstrated the limited utility of single serum ADAM12 measurements as an early diagnostic biomarker for tubal EP.

### Limitations to the study

A limitation of the study is the relatively small sizes of the patient groups, and these may not accurately represent the actual biomarker levels during the development and onset of tubal EP. Thus additional large-scale studies are needed to determine the changes in selected biomarker levels before onset of tubal EP to validate their utility in diagnosing tubal EP. Because it is ethically impossible to obtain tissues from early IUP and post-surgery EP patients, another limitation is that our study lacks data using Western blotting analysis and immunohistochemistry for determining VEGF-A, PIGF, and ADAM12 expression levels in these patients.

## Conclusions

In summary, we have demonstrated that individual measurements of serum E2 or VEGF-A levels are strongly related to early pregnancy outcomes for women with IUP and EP, and pregnancy-associated E2 and VEGF-A levels may serve as biomarkers for the early diagnosis of tubal EP. Although our study also highlights the potential use of combined E2/VEGF-A or E2/PIGF measurements as promising diagnostic biomarkers for tubal EP, the limitations of our study warrant further investigation. Further studies using two relatively large prospective cohorts with well-defined and gestational age-matched tubal EP and IUP are warranted to confirm the clinical relevance of these correlations in women with tubal EP.

## Abbreviations

EP: Ectopic pregnancy;IUP: Intrauterine pregnancy;E2: 17β-estradiol;P4: Progesterone;T: Testosterone;β-hCG: Beta-human chorionic gonadotropin;VEGF-A: Vascular endothelial growth factor-A;PIGF: Placental growth factor;ADAM12: A distintegrin and metalloprotease protein 12

## Competing interests

The authors report no conflict of interest.

## Authors’ contributions

SZ, XL, YF, SS, JL, and EE participated in the recruitment of patients, conducted the experiments, performed statistical analysis of the data, and made the tables and figures. HB provided clinical insights, interpreted the data, and edited the paper. RS designed, supervised and coordinated the study and drafted the manuscript. Each author has taken public responsibility for appropriate portions of the content and all authors have read and approved the final manuscript.
